# Higher Chondrogenic Potential of Extracellular Vesicles Derived from Mesenchymal Stem Cells Compared to Chondrocytes-EVs In Vitro

**DOI:** 10.1155/2021/9011548

**Published:** 2021-12-13

**Authors:** Maryam Hosseinzadeh, Amir Kamali, Samaneh Hosseini, Mohamadreza Baghaban Eslaminejad

**Affiliations:** ^1^Department of Stem Cells and Developmental Biology, Cell Science Research Center, Royan Institute for Stem Cell Biology and Technology, ACECR, Tehran, Iran; ^2^Faculty of Sciences and Advanced Technologies in Biology, University of Science and Culture, Tehran, Iran; ^3^Department of Cell Engineering, Cell Science Research Center, Royan Institute for Stem Cell Biology and Technology, ACECR, Tehran, Iran

## Abstract

The inability of cartilage to self-repair necessitates an effective therapeutic approach to restore damaged tissues. Extracellular vesicles (EVs) are attractive options because of their roles in cellular communication and tissue repair where they regulate the cellular processes of proliferation, differentiation, and recruitment. However, it is a challenge to determine the relevant cell sources for isolation of EVs with high chondrogenic potential. The current study aims to evaluate the chondrogenic potential of EVs derived from chondrocytes (Cho-EV) and mesenchymal stem cells (MSC-EV). The EVs were separately isolated from conditioned media of both rabbit bone marrow MSCs and chondrocyte cultures. The isolated vesicles were assessed in terms of size, morphology, and surface marker expression. The chondrogenic potential of MSCs in the presence of different concentrations of EVs (50, 100, and 150 *μ*g/ml) was evaluated during 21 days, and chondrogenic surface marker expressions were checked by qRT-PCR and histologic assays. The extracted vesicles had a spherical morphology and a size of 44.25 ± 8.89 nm for Cho-EVs and 112.1 ± 10.10 nm for MSC-EVs. Both groups expressed the EV-specific surface markers CD9 and CD81. Higher expression of chondrogenic specified markers, especially collagen type II (COL II), and secretion of glycosaminoglycans (GAGs) and proteoglycans were observed in MSCs treated with 50 and 100 *μ*g/ml MSC-EVs compared to the Cho-EVs. The results from the use of EVs, particularly MSC-EVs, with high chondrogenic ability will provide a basis for developing therapeutic agents for cartilage repair.

## 1. Introduction

Articular cartilage (AC) does not have blood vessels and possesses low cell density; therefore, even minor injuries can lead to disorders such as osteoarthritis (OA) [1]. Traditional therapeutic methods that include drug therapy and surgery are unable to completely heal the injured cartilage [2, 3]. Among various novel approaches, administration of extracellular vesicles (EVs) is under consideration as a cell-free and noncellular approach. EVs overcome the challenges attributed to stem cell therapy such as immunogenicity and tumorigenesis [4, 5].

EVs are vesicles that have lipid bilayer membranes, which can be isolated from a variety of cells as well as body fluids such as serum, saliva, cerebrospinal fluid, and synovial fluid [6, 7]. The results of the studies show that the contents of EVs are representative of the cells from which they are isolated [8–10]. Among various cell sources, EVs derived from mesenchymal stem cells (MSC-EVs) have been extensively used to repair different tissues, including the heart and kidneys [11, 12]. Several research groups have also studied the effect of EVs isolated from different MSC sources on cartilage tissue repair. Zhang et al. evaluated the regenerative ability of EVs isolated from human embryo-derived MSCs on osteochondral defects. They observed the formation of subcartilaginous bone-like tissue and hyaline cartilage tissue after 12 weeks in the knee that was treated with EVs, whereas fibrocartilage formed in the control group [13]. Zhang et al. investigated the effect of MSC-derived EVs on cell growth and migration, immune response, and ECM synthesis through s-ACAN and collagen type II (COL II) at the osteochondral defects. Their findings confirmed an increase in growth and migration of chondrocytes in groups treated with EVs [14]. It has been demonstrated that embryonic stem cell-induced MSC-derived exosomes can mitigate cartilage destruction and matrix degradation in destabilized medial menisci (DMM) in C57BL/6 mouse models of OA. Their results indicated a reduction in ADAMTS5 expression in the presence of IL-1*β* and an elevation in COL II synthesis [15]. Genetically modified cells were also used to enrich the EV content. The results of these studies revealed that any modifications to cell sources would impact the EV contents and confirmed the role of the microenvironment on determining the EV content [16, 17]. In a study performed by Tao's group, EVs isolated from manipulated synovial MSCs led to increased proliferation and differentiation into chondrocytes *in vitro*. These EVs could also prevent the progression of OA in a rat model [18]. Although the positive effect of EVs on cartilage regeneration has been addressed, researchers have yet to clarify whether EVs isolated from different cell sources have similar chondrogenic capacities.

Chondrocytes are another cell source in cartilage tissue in addition to chondrocyte progenitor cell (CPC) and BMSCs. According to Wang et al., both CPCs and chondrocytes are more capable of chondrogenesis than BMSCs [19]. Thus, chondrocytes provide a convenient cell source for EV isolation, and it is expected that they can repair cartilaginous tissue because of their location in the cartilaginous niche. However, there has been little investigation of chondrocyte-isolated EVs. In an initial study, Chen et al. injected exosomes derived from rabbit chondrocytes and MSCs into a CPC construct to stimulate ectopic cartilage regeneration in subcutaneous spaces in mice. They investigated the effect of MSC- and chondrocyte-exosomes on the proliferation and differentiation of CPCs *in vitro*. According to this study, exosomes derived from chondrocytes stimulated CPC proliferation and increased the expressions of chondrogenic markers [20]. More recently, Ma et al. investigated the impact of human chondrocytes-EVs on chondrogenic differentiation of umbilical cord-derived stem cells (HUCMSCs). They found that chondrocyte-EVs promoted chondrogenesis of HUCMSCs compared to an untreated group [21]. Although these studies attempted to show the chondrogenic potential of chondrocyte-EVs, the difference between chondrogenic potential of MSC-EVs and chondrocyte-EVs on MSCs should be addressed. Additionally, the effective concentration of EVs is one challenge that should be examined in greater detail.

Therefore, we conducted the current study to evaluate and compare the chondrogenesis capability of MSCs and chondrocyte-isolated EVs on bone marrow-derived MSCs *in vitro*. We also sought to determine the effective concentration of EVs for cartilage regeneration. The EVs were isolated from MSCs and chondrocytes, and we compared their characteristics. Finally, a cell micromass was established as an *in vitro* chondrogenic culture model to assess the chondrogenic potential of these isolated EVs.

## 2. Materials and Methods

### 2.1. Mesenchymal Stem Cell (MSC) Isolation, Culture, and Characterization

MSCs were aspirated from the bone marrow of White New Zealand male rabbits. The isolated MSCs were cultured in 75 cm^2^ T-flasks that contained DMEM media supplemented with 10% fetal bovine serum (FBS) and 1% penicillin/streptomycin at 37°C and 5% CO_2_ [22]. The medium was changed twice weekly until the cells reached 80% confluency. Passage-3 cells were used for further experiments. The expressions of surface markers CD34 (CD34 Antibody, bs-2038R, Biocompare, USA), CD19 (CD19 Antibody (FITC), HIB19, eBioscience, USA), CD45 (CD45 Antibody, orb435180, Biorbyt, USA), CD90 (CD90 Antibody, MCA47FT, Bio-Rad, USA), CD105 (CD105 Antibody [SN6] (FITC), GTX11415, GeneTex, USA), and CD73 (CD73 Antibody, bs-4834R, biossusa, USA) were assessed by flow cytometry to confirm the mesenchymal phenotype of the isolated cells. These isolated cells were also characterized based on their differentiation ability into osteoblasts and adipocytes. For this purpose, 1 × 10^5^ cells were cultured in each well of a 6-well culture plate. The basic culture media was replaced by osteogenic or adipogenic induction media when the cells reached 70–80% confluency. The wells that contained basic media were considered the control group. The cells were stained with alizarin red S (ARS) and oil red O on day 21 to evaluate the osteogenic and adipogenic differentiation potential, respectively. We used a micromass culture system to induce chondrogenic differentiation of the isolated cells. Briefly, 2.5 × 10^5^ passage-3 rabbit bone marrow MSCs were centrifuged at 1200 g for 5 min to form cell pellets for micromass formation in a chondrogenic medium. The cells were maintained in this medium for 21 days at 37°C and 5% CO_2_, with twice-weekly medium changes. Chondrogenic differentiation was assessed by safranin O and toluidine blue staining of the pellet sections.

### 2.2. Chondrocyte Isolation and Culture

Cartilage tissues were dissected from the knees of the rabbits in accordance with the standard operating procedures approved by the Animal Care and Ethics Committee at Royan Institute, Tehran, Iran. The specimens were transferred to the laboratory where the cartilage layer was aseptically dissected from the knee condyle by a surgical razor. Next, the cartilages were cut into 2–3 mm pieces and kept overnight in a 0.8% collagenase solution at 37°C. The isolated chondrocytes were collected by centrifugation at 1200 rpm for 5 min and subsequently transferred to a cell culture plate. The culture media was changed every three days, and the cells were passaged weekly. We used cells at passage two for further experiments.

### 2.3. Extracellular Vesicle (EV) Isolation

The EVs were separately isolated from the culture media that contained the MSCs or chondrocytes. The MSCs were cultured in basic DMEM culture media supplemented with 15% EV-free FBS and 1% penicillin/streptomycin. Chondrocytes were cultured in basic media that contained 10% EV-free FBS and 1% penicillin/streptomycin. The condition media of both cells were collected every other day for one week.

We used ultracentrifugation to collect the EVs from the conditioned medium. First, the media were centrifuged at 1500 × g for 15 min to remove the nonviable cells, followed by centrifugation at 20 000 × g for 15 min. Then, the supernatants were centrifuged twice at 100 000 × g for 2 h, after which the supernatant was discarded. The precipitate that contained the EVs was suspended in sterile phosphate-buffered solution (PBS) and kept at -80°C until further analysis. The MSC-EVs represented EVs that were isolated from MSCs, and the Cho-EVs were isolated from chondrocytes.

To reduce the batch effect, we cultured the cells, collected all media, and isolated EVs from the pool of conditioned medium. Next, the isolated EVs were characterized and aliquoted and just one EV batch was used for all experiments.

### 2.4. Extracellular Vesicle (EV) Characterization

The average diameter of the isolated EVs was determined by dynamic light scattering (DLS) measurement with a Zetasizer (Malvern Instrument, UK). We diluted the samples 1 : 1000 in PBS to a total volume of 1 ml. Next, three measurement runs were performed with a refractive index of 1.331, viscosity of 0.9, and a temperature of 25°C. Data were analyzed by Malvern Zetasizer Software (Malvern Instrument, UK).

The morphology of EVs was characterized using a scanning electron microscope (SEM). Fresh EVs were first diluted in PBS, and 5 *μ*l of the prepared mixture was placed on a glass slide. Subsequently, the samples were coated with spray gold (SCD005, Bal-Tec, Switzerland) and images were taken by the SEM (XL30, Philips, The Netherlands).

Calcein green fluorescent dye (calcein, AM, c3099, Invitrogen, USA) was used to evaluate MSC uptake of the EVs. We incubated 10 *μ*g/ml of exosomes in 1 × 10^–6^ M dye for 30 min at 37°C. After resuspension in PBS, the exosomes were centrifuged (100 000 × g for 1 h). The seeded MSCs were incubated at 37°C until they became 60% confluent; then, they were labeled with PKH26 (PKH26-Red, PKH26GL, Sigma, Germany) overnight. Subsequently, they were incubated overnight with calcein-stained exosomes. In order to visualize their nuclei, the cells were stained with DAPI (aqueous DAPI (Fluoroshield) ab104139, Abcam, UK). The stained and fixed cells were subsequently imaged.

### 2.5. Western Blot Analysis

The EV concentration was quantified by the BCA Protein Assay Kit (Millipore, Germany) according to the manufacturer's instructions. A total of 15 *μ*g of EV protein from each sample were suspended in sample loading buffer solution at an equal volume, sonicated, and heated to 95°C for 5 min. The samples were separated on 12% polyacrylamide gels and transferred onto an electrophoretic (Bio-Rad) polyvinyl difluoride (PVDF) membrane (Millipore, Germany) (semidry method, 120 V, and 75 min). The membrane was blocked with 5% bovine serum albumin (BSA) for one hour at room temperature, followed by incubation overnight at 4°C with primary antibodies specific to CD81 (EX203, Cell Guidance Systems, UK) and CD9 (GTX76182, GeneTex, USA), and the negative organelle marker, calnexin (1 : 500, sc-11397, Santa Cruz Biotechnology, USA). The membranes were subsequently incubated with goat anti-mouse HRP-conjugated secondary antibodies (1 : 5000, ab6789, Abcam, US) for 2 h at room temperature. After three washes with 1x TBST, we added enhanced chemiluminescence (ECL) to the blots and the protein bands were visualized by a chemiluminescence device (Uvitec, Cambridge, UK).

To evaluate the expression level of chondrogenic proteins in the isolated EVs, we also performed wet western blot analysis with the COL II (COL2A1, 250484, Abbiotec, USA), aggrecan (ACAN) (NB600-504, Novus Biologicals, USA), and COL X (COL10A, ab58632, Abcam, UK) primary antibodies. The labeled protein bands were analyzed with ImageJ analysis software.

### 2.6. Chondrogenic Potential of the Extracellular Vesicles (EVs)

To evaluate the chondrogenic potential of the EVs, 2.5 × 10^5^ passage-3 MSCs were centrifuged at 1200 g for 5 min to form cell pellets for micromass formation. Micromass facilitates cell-cell interactions by providing a three-dimensional environment. Micromass were cultured in a chondrogenic medium (CM) that contained high-glucose DMEM supplemented by 10 ng/ml transforming growth factor-*β*1 (TGF-*β*1, Sigma, Germany), 1% insulin-transferrin-selenium (ITS, Gibco, USA), 10^−7^ M dexamethasone (Sigma), 50 *μ*g/ml ascorbate-2-phosphate, and 1% nonessential amino acids in the presence of different concentrations (50, 100, and 150 *μ*g/ml) of EVs for 21 days. The rabbit bone marrow MSC cell pellets that were cultured in CM without EVs were considered to be the negative control groups. All the experiments were conducted in three independent biological replicates.

### 2.7. qRT-PCR

qRT-PCR was used to analyze the expressions of genes related to chondrogenesis (*Acan*, *Col II*, and *Sox9*), and *Col X* (hypertrophy marker). RNA extraction and simultaneous cDNA synthesis from the cell micromass were performed with a cell-to-cDNA kit. The qRT-PCR interactions were conducted with a Maxima SYBR Green/ROX qPCR Master Mix (Yekta Tajhiz, Iran) in an Applied Biosystems Step One Plus Real-time PCR system. The specified strip was set on the ice according to the number of samples. The samples were collected from three independent biological replicates. Each sample was repeated twice, and two negative controls were considered for each primer.  Table 1 shows the primers used for this assessment.

### 2.8. Histology and Immunohistochemistry

Cell aggregates were fixed in 4% paraformaldehyde at 4°C and embedded in paraffin. Next, they were cut into 6 *μ*m thick sections, dehydrated with alcohol, and stained with safranin O and toluidine blue (TB). The slides were photographed by an optical microscope (Olympus BX51; Olympus, Tokyo, Japan).

Immunohistochemistry staining was used to characterize the density of the chondrogenic proteins of COL II and ACAN. After 21 days of culture, the aggregates were fixed in 4% paraformaldehyde at 4°C and 6 *μ*m thick sections were prepared. After dehydration with alcohol, antigen retrieval was performed with 0.05% trypsin for 30 min at 37°C. The sections were blocked with 1.5% goat serum in PBS and treated overnight at 4°C with rabbit anticollagen type II (250484, Abbiotec, USA) and ACAN (NB600-504, Novus Biologicals, USA) primary antibodies. Next, they were incubated for one hour with anti-mouse (A2554, Sigma-Aldrich, Germany) and anti-rabbit (ab97051, Abcam, UK) secondary antibodies. Images were acquired by an optical microscope (Olympus BX51; Olympus, Tokyo, Japan), and the density of the staining was assessed by Image-Pro Plus 6 software.

### 2.9. Statistical Analysis

Data were analyzed by *t*-test for two groups, and the results were compared between each group using one-way ANOVA analysis with GraphPad Prism software. Data are presented as mean ± SEM. The results were considered statistically significant at *p* < 0.05.

## 3. Results

### 3.1. Isolation, Proliferation, and Characterization of Mesenchymal Stem Cells (MSCs)

**Figure 1:**
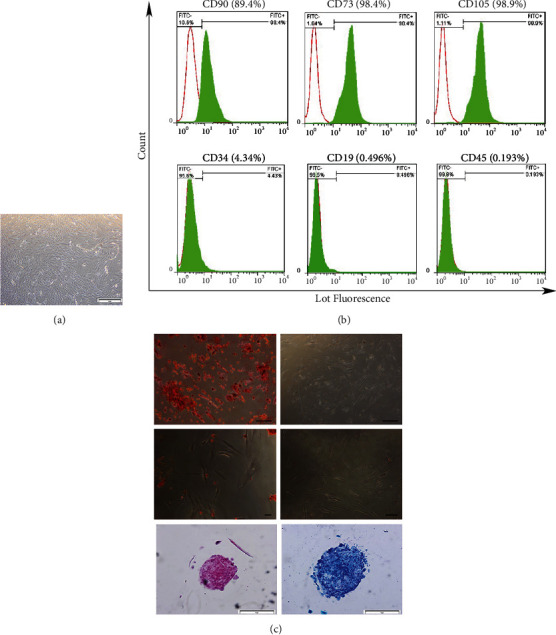
Characterization of mesenchymal stem cells (MSCs) derived from rabbit bone marrow. (a) Primary culture of cells derived from bone marrow. (b) Flow cytometry analysis of the expressions of the MSC surface markers. (c) Differentiation of multipotent MSCs into (a) osteocytes and (b) the control group after 21 days. Differentiation of multipotent MSCs into (c) adipocytes and (d) the control group after 21 days. Differentiation of MSCs into chondrocytes after 21 days; safranin O (e) and toluidine blue staining (f).

We isolated and expanded plastic-adherent cells that had a spindle-like shape. These cells proliferated and formed discrete colonies (Figure 1(a)). The expression levels of CD90 and CD34 surface markers were evaluated by flow cytometry. The results showed that the majority of bone marrow-derived cells were positive for CD90 (89.4%), CD 73 (98.4%), and CD 105 (98.9%). They were also negative for CD 19 (0.496%) and CD 45 (0.193%). Only 4.43% of the isolated cells expressed CD34, a hematopoietic marker (Figure 1(b)). The MSCs gave rise to osteogenic, adipogenic, and chondrogenic lineages when cultured in their related induction media. ARS results confirmed the osteogenic potential of these MSCs by the presence of bone-like nodules in the extracellular matrix (ECM) (Figure 1(c), A and B). Similarly, we observed the accumulation of intracellular oil droplets in the MSCs (Figure 1(c), C and D). We sought to verify that the MSCs differentiated into a chondrogenic lineage. After 21 days in a chondrogenic medium, the cells were stained with safranin O and toluidine blue in order to detect the presence of proteoglycans (Figure 1(c), E and F).

### 3.2. Characterization of Isolated Extracellular Vesicles (EVs)

**Figure 2:**
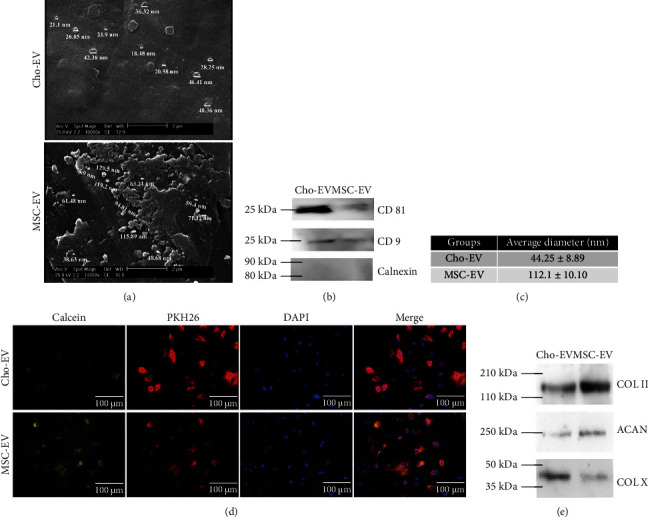
Characterization of extracellular vesicles (EVs) derived from chondrocytes (Cho-EVs) and mesenchymal stem cells (MSCs) (MSC-EVs). (a) Scanning electron microscope (SEM) images of Cho-EVs and MSC-EVs. (b) Western blot analysis of specific EV surface marker expression, including CD9 and CD81 and calnexin. (c) Particle size distribution measured by dynamic light scattering (DLS). (d) Uptake of EVs by calcein staining. (e) Western blot analysis of EV proteins, including COL II, ACAN, and COL X. kDa: kilodalton.

EVs isolated from MSCs and chondrocytes were characterized in terms of size, morphology, and EV-related markers. SEM images confirmed the spherical morphology of the isolated EVs for both MSCs and chondrocytes (Figure 2(a)). Western blot analysis indicated the presence of the specific surface proteins CD81 and CD9 in the samples. The Cho-EVs had a higher level of CD81 expression compared to MSC-EVs (Figure 2(b)). Isolated EVs were also negative for calnexin. DLS assessment verified that the Cho-EVs were 44.25 ± 8.89 nm in diameter and the MSC-EVs were 112.1 ± 10.10 nm in diameter (Figure 2(c)).  Figure 2(d) shows the internalization of the calcein-labeled EVs by MSCs after 24 h. Fluorescence microscopy images revealed the presence of calcein-labeled EVs in the cytoplasm of the MSCs, which confirmed that the isolated EVs were successfully internalized by the MSCs.

The western blot analysis of EV's chondrogenic and hypertrophic markers in isolated EVs is shown in Figure 2(e). Interestingly, both MSC-EVs and Cho-EVs contained COL II and ACAN proteins. However, the expression level of ACAN was lower than COL II in both EVs (*p* < 0.05). MSC-EVs had a higher level of COL II compared to Cho-EVs (*p* < 0.05). The similar level of ACAN was observed in both groups. The hypertrophic marker of COL X was also detected in both MSC-EVs and Cho-EVs, but its level was higher in Cho-EVs (*p* < 0.05).

### 3.3. The Effect of Isolated Extracellular Vesicles (EVs) on Chondrogenic Differentiation of Mesenchymal Stem Cells (MSCs)

**Figure 3:**
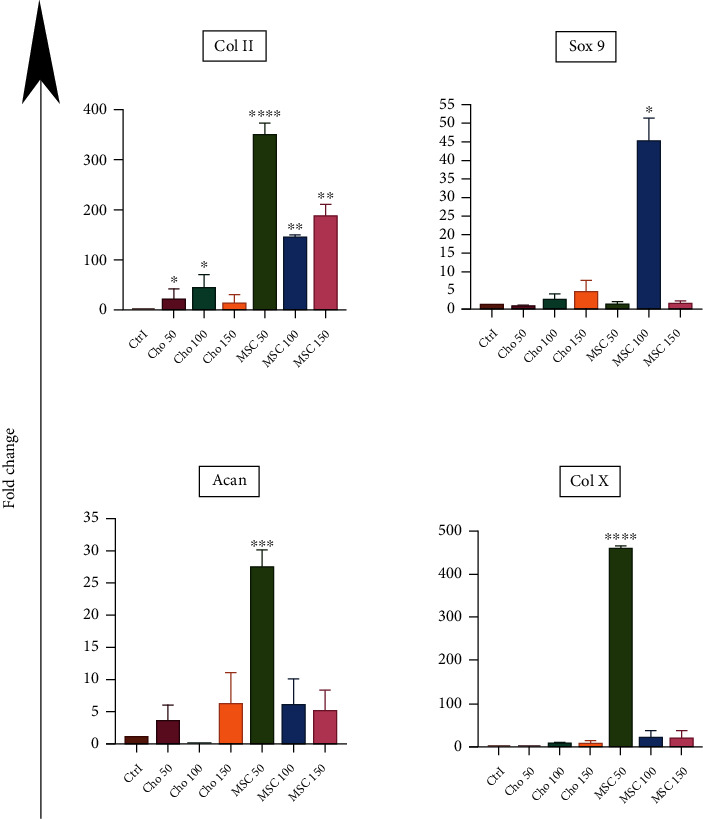
qRT-PCR analysis of chondrogenic-related genes. Histogram shows the qRT-PCR analysis of type II collagen (*Col II*), *Sox 9*, aggrecan (*ACAN*), and *Col X* in a cell micromass culture in the presence of different concentrations (50, 100, or 150 *μ*g/ml) of extracellular vesicles (EVs) after 21 days. ^∗^*p* < 0.05; ^∗∗^*p* < 0.01; ^∗∗∗^*p* < 0.001; and ^∗∗∗∗^*p* < 0.0001 compared to the control group.

Figure 3 shows the expression level of specific chondrogenic genes including *Col II*, *Acan*, *Sox 9*, and *Col X* analyzed using qRT-PCR. The gene expression level of *Col II* significantly increased in the groups that received MSC-EVs, compared with the Cho-EV group and the control group. The cell pellets that had 50 *μ*g/ml MSC-EVs expressed a higher level of the *Col II* gene compared with the 100 *μ*g/ml MSC-EV and 150 *μ*g/ml MSC-EV groups (*p* < 0.0001; *p* < 0.01). In the groups that received Cho-EVs, we observed higher expression levels of *Col II* in the 50 and 100 *μ*g/ml Cho-EV groups compared with the control group (*p* < 0.05). The *Acan* gene significantly increased in the 50 *μ*g/ml MSC-EV group (*p* < 0.001). Although we observed an increased expression level of *Acan* in the other groups compared to the control group, these increases were not statistically significant. The *Sox 9* gene had the highest expression in the MSCs treated with 100 *μ*g/ml MSC-EV compared with the other groups (*p* < 0.05). The other groups showed slight increases in *Sox9* gene expression compared to the control group. *Col X* expression, a hypertrophic marker for chondrogenic differentiation, was also assessed. We did not observe any significant increase in the expression of *Col X* in any of the Cho-EV concentrations compared to the control group. Among the MSC-EV groups, MSCs exposed to the 50 *μ*g/ml MSC-EV had the highest expression level of *Col X*; a very slight increase in *Col X* was observed in the 100 and 150 *μ*g/ml MSC-EV groups (*p* < 0.0001).

**Figure 4:**
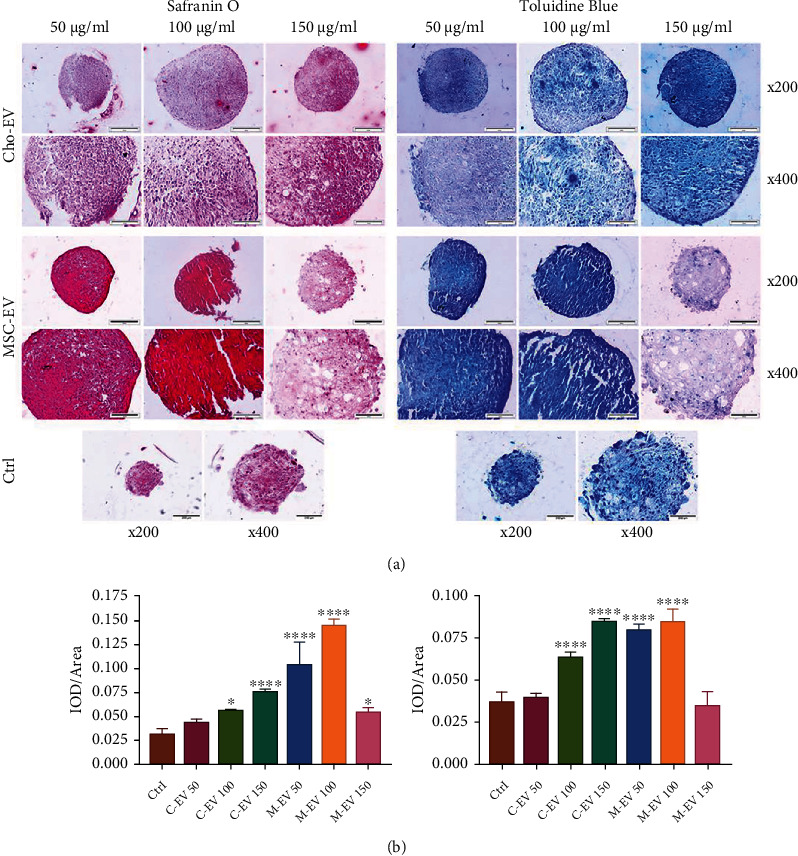
Histological analysis. (a) Safranin O and toluidine blue (TB) staining of mesenchymal stem cells (MSCs) that differentiated into chondrocytes in the presence of extracellular vesicles (EVs) derived from chondrocytes (Cho-EV) and MSCs (MSC-EV) after 21 days. (b) Histograms show the intensity of (a) red and (b) purple colors among all groups by safranin O and TB staining, respectively. ^∗^*p* < 0.05; ^∗∗∗^*p* < 0.001; and ^∗∗∗∗^*p* < 0.0001 compared to the control group.

Glycosaminoglycan- (GAG-) rich sites turned red after safranin O staining, which verified the efficient chondrogenic differentiation of MSCs in groups that contained EVs compared to the control group. The MSCs treated with 100 *μ*g/ml MSC-EV, 50 *μ*g/ml MSC-EV, and 150 *μ*g/ml Cho-EV had the highest amounts of GAGs, respectively (*p* < 0.0001), which led to better differentiation among all of the groups. We observed a similar intensity in red color in the MSCs that received 150 *μ*g/ml MSC-EV and 100 *μ*g/ml Cho-EV (*p* < 0.05); however, there was no significant increase detected in the 50 *μ*g/ml Cho-EV group compared to the control group (Figure 4(a)). The pericellular matrix in the lacuna became purple after TB staining, which indicated their metachromatic properties. As can be seen in Figure 4(a) and the histogram in Figure 4(b), the 150 *μ*g/ml Cho-EV, 50 *μ*g/ml MSC-EV, and 100 *μ*g/ml MSC-EV groups showed similar intensities in the purple color and the highest chondrogenic differentiation among all groups (*p* < 0.0001). We observed that metachromasia in the cell pellets exposed to 100 *μ*g/ml Cho-EV was statistically significant compared to the control group (*p* < 0.0001). There were no differences between the 50 *μ*g/ml Cho-EV and 150 *μ*g/ml MSC-EV groups compared to the control group.

**Figure 5:**
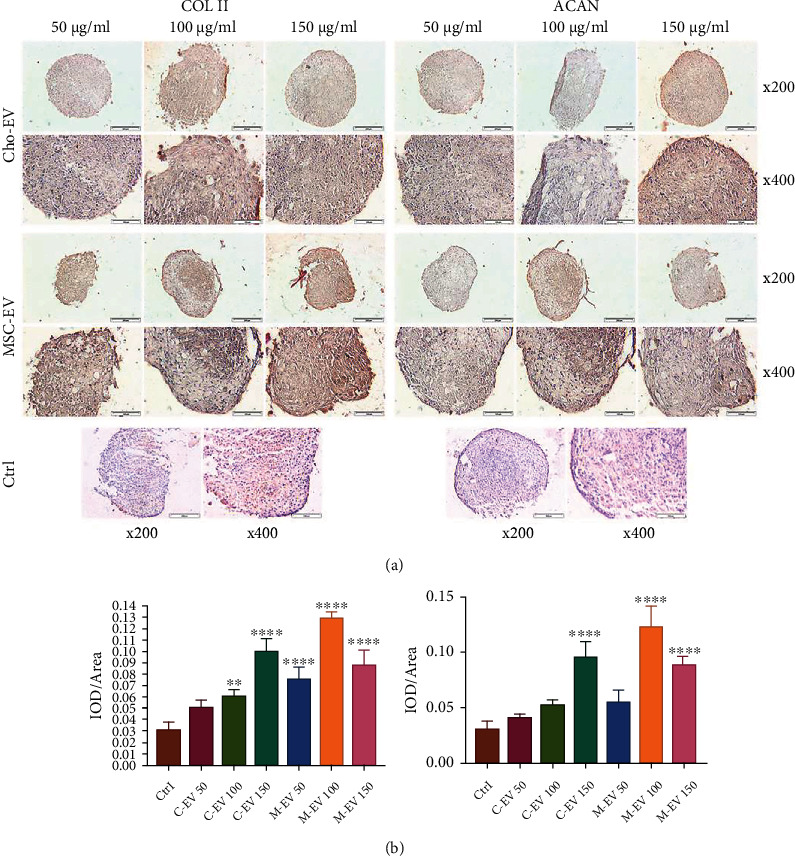
Immunohistochemical analysis of chondrogenic proteins. (a) IHC staining of type II collagen (Col II) and aggrecan (ACAN). (b) Histograms show the intensity of Col II and ACAN proteins expressed in mesenchymal stem cells (MSCs) that differentiated into chondrocytes in the presence of extracellular vesicles (EVs) derived from chondrocytes (Cho-EV) and MSCs (MSC-EVs) after 21 days. ^∗^*p* < 0.05; ^∗∗^*p* < 0.01; ^∗∗∗^*p* < 0.001; and ^∗∗∗∗^*p* < 0.0001 compared to the control group.

The results of immunohistochemical staining and related histograms of differentiated cell micromasses in the presence of different concentrations of Cho-EVs and MSC-EVs against the COL II and ACAN proteins are shown in Figures 5(a) and 5(b). There was significant expression of the COL II protein in the groups that contained MSC-EVs (all concentrations) and Cho-EVs (100 and 150 *μ*g/ml) compared to the control group (*p* < 0.0001; *p* < 0.01). A comparison of COL II expression levels between the MSC-EVs and the Cho-EVs indicated that the group that contained MSC-EV had higher expression levels. The increased expression level of the ACAN protein showed the same trend as COL II in all groups; the increase in 50 *μ*g/ml Cho-EV, 100 *μ*g/ml Cho-EV, and 150 *μ*g/ml MSC-EV was not statistically significant compared to the control group.

## 4. Discussion

The lack of self-repair capability in cartilage tissue necessitates the use of therapeutic approaches to prevent the injury and disease progression [23]. Recently, EVs have emerged as a therapeutic agent to promote tissue repair without the difficulties associated with cell therapy. In the present study, we evaluated the chondrogenic potential of EVs isolated from MSCs and chondrocytes to find an ideal cell source that could be used to isolate EVs for cartilage tissue regeneration.

We successfully isolated MSCs from rabbit bone marrow and confirmed their characteristics according to their fibroblastic and spindle-like appearance, plastic-adherent ability, expression of specific cell surface markers, and differentiation potential into mesenchymal lineages. Analysis of cell surface markers revealed that isolated cell populations expressed the mesenchymal cell marker CD90, CD73, and CD105 and were negative for CD34, CD19, and CD45 which supported findings from previous studies [24]. We examined the ability of the isolated cells to differentiate into a skeletal lineage. The ability of MSCs from the bone marrow to differentiate into a mesodermal lineage is well-documented [25]. In this study, the osteogenic, adipogenic, and chondrogenic abilities of the isolated cells were supported by the appearance of mineralized nodules, oil droplets, and proteoglycan-rich areas that stained positively after the addition of alizarin red, oil red O, and specific chondrogenic staining, respectively.

Analysis of the isolated EV characteristics showed that the MSC-EVs were approximately 112 nm in size. These results agreed with other studies that characterized EVs from MSC sources [26, 27]. In comparison, the Cho-EVs were smaller than the MSC-EVs. A recent study reported that EVs ranged from 30 to 200 nm for those isolated from rabbit chondrocytes and bone marrow MSCs. Our results were also within this range [20]. However, they found no difference between both EVs in terms of size and diameter. This discrepancy might be allocated to the different EV isolation techniques. The diameter of the isolated EVs agreed with the results obtained from SEM micrographs. Moreover, both groups expressed the protein surface markers, CD81 and CD9, and were negative for calnexin in accordance with standard definitions of EVs and the International Society of Extracellular Vesicles (ISEV) [28, 29].

To address the chondrogenic potential of MSC-EVs and Cho-EVs, we first evaluated the expression levels of chondrogenic-related genes *Col II, Acan*, and *Sox9*, and a hypertrophic marker of *Col X*. *Col II* and *Acan* are important genes in the cartilage ECM, and their upregulation indicates differentiation into a chondrocyte lineage. COL II is the predominant component of the cartilage matrix. COL II with other proteins and proteoglycans provide complex extracellular substrates for the attachment of chondrocytes, extracellular matrix (ECM) molecules, and growth factors [30]. It is an important extracellular signaling molecule that can promote chondrocyte proliferation, differentiation, and ECM deposition [31]. Our results are consistent with previous studies that have shown that *Acan* and *Col II* were expressed as chondrogenic markers in the late stage of chondrogenic differentiation [21, 32]. Herein, a higher expression level of these genes in the MSC-EV group revealed that MSC-EVs were more efficient than the Cho-EVs for chondrogenesis. *Sox9* encodes one of the important transcription factors in differentiation into the chondrocyte lineage, which induces the expression of other genes (*Col II* and *Acan*) involved in this pathway [33, 34]. We observed the expression of this gene, particularly the 100 *μ*g/ml concentration, in the MSC-EV containing groups. Previous studies have reported the increase of cartilage-specific genes in the groups that contained MSC-derived EVs, which confirmed the results of the present study. On the other hand, the expression level of genes in the MSCs exposed to Cho-EVs was lower than the MSC-EV groups, which contrasted with the results of a study reported by Chen et al. The expression level of genes in their study for the formation of ectopic cartilage *in vivo* was reported to be the same for both chondrocyte- and MSC-derived EVs [20]. The difference between that study and the present study was the evaluation of the differentiation potential of cartilage *in vitro*. In a study by Chen et al., there was no difference in size and the expression levels of the surface markers were reported for both EV groups. However, the isolated EVs in the present study differed in both the size and expression levels of the surface markers. This result could lead to a difference in the functionality of the two EVs. The expression of the *Col X* hypertrophic marker was also evaluated in both the Cho-EV and MSC-EV groups. The *Col X* gene is expressed in hypertrophic chondrocytes and is an important marker for endochondral ossification, which involves a programmed process of chondrocyte hypertrophy. About 45% of the expressed collagens of hypertrophic chondrocytes consist of this type [35, 36]. Hypertrophy is one challenge for using chondrocytes that are differentiated from bone marrow MSCs *in vitro* [37]; this differentiation leads to an unstable and unsuitable chondrogenic phenotype for therapeutic approaches. The *Col X* gene was highly expressed in groups that received 50 *μ*g/ml of MSC-EVs. However, an increase in EV concentration resulted in the downregulation of this gene. A recent study reported that a certain threshold of *Col X* expression was necessary for matrix formation during MSC-mediated chondrogenesis. Once *Col X* knockdown was more than 80%, major ECM components, including *Col II* and *Acan*, significantly downregulated [38, 39]. On the other hand, *Col X* expression was lower in the Cho-EV groups compared to the MSC-EV groups. Studies have shown that MSC-EVs have the potential for hypertrophic cartilage formation [20]. Thus, it may be rational to expect that the expression of this gene would be higher in the MSC-EV groups in comparison with the Cho-EV groups.

Histological and immunohistochemical analyses were also performed. The tissue sections showed that the cells had a normal cartilage-like pattern in the cell micromass. The cells located in the outer layer of the micromass formed a perichondrium-like layer and surrounded the inner cells. The cartilage matrix contained GAG. This was attributed to the presence of carbohydrates and high amounts of negatively charged sulfate groups that, in conjunction with GAG, resulted in the metachromatic properties seen with TB staining. The pericellular matrix of cells in the lacuna was stained purple (basophilic). The cells that were present in the lacuna-like cavities were spherical, whereas cells that were located in the marginal areas were relatively elongated. We observed chondrogenic differentiation in the MSC-EV-containing groups, which agreed with the qRT-PCR results. Overall, the groups that received EVs particularly MSC-EVs showed higher chondrogenic differentiation compared to the control group. Notably, we believe the reason behind the downregulation of chondrogenic genes and decrease of proteoglycans in a group that received 150 *μ*g/ml of MSC-EVs is attributable to the size of EVs. Since MSC-EVs had a larger size compared to Cho-EVs, the higher concentration of large MSC-EVs may prevent the cells to be dense sufficiently. In the process of cartilage differentiation, the first major factor is the density of MSCs, which occurs with the help of growth factor and expression of *Sox9* [40, 41]. Dense and contracted MSCs during 21 days of culture promote the production of proteoglycans and *Col II* which has not occurred in 150 *μ*g/ml of the MSC-EV group.

We sought to explain why higher chondrogenic differentiation occurred in the MSC-EV-containing groups than Cho-EVs. The contents of EVs with regard to chondrogenic markers were characterized. Surprisingly, both MSC-EVs and Cho-EVs contained COL II, ACAN, and COL X. The presence of COL II, ACAN, and COL X in the EVs was believed to be the rationale behind their chondrogenic potential. The improved chondrogenic ability of MSC-EVs may allocate to their higher level of COL II. As mentioned above, COL X expression occurred within the first few days of the chondrogenesis process. It has also been reported that there is a close relation between COL II and COL X, which their expression almost simultaneously occurs during early chondrogenic differentiation [39]. Therefore, the existence of these markers in isolated EVs would be rational. Although the reason MSC-EVs and Cho-EVs have similar contents is unclear, it could be related to their intrinsic properties. More recently, Fabre et al. reported that chondrocytes and MSCs share similar biological characteristics as evidenced by flow cytometry analysis of classical MSC markers and BMMSCs represented higher chondrogenic potential among various sources of MSCs [42].

These results suggest that MSC-isolated EVs that carry chondrogenic markers would be a better choice for chondrogenic differentiation compared to chondrocyte-isolated EVs.

## 5. Conclusion

Our results demonstrated that EVs from chondrocytes and MSCs could enhance the chondrogenesis of MSCs in a cell micromass culture that is induced by TGF-beta signaling. This *in vitro* study showed that both MSCs and chondrocyte-derived EVs affected chondrogenic differentiation; however, EVs derived from MSCs were more efficient, especially at the 100 *μ*g/ml concentration. We demonstrated that EVs could modulate chondrogenic differentiation via *Col II* and *Sox9* expression and reduce the hypertrophic marker of *Col X*. MSC-EVs demonstrated a beneficial effect on chondrogenicity by modification of the synthesis of the chondrogenic ECM and specific genes and proteins. This indicates a potential use for these EVs as a new tool for cartilage repair because of their ability to enhance cartilage differentiation. Further experiments are necessary to clearly elucidate the molecular mechanisms implicated in the acceleration of chondrogenesis related to EVs.

## Figures and Tables

**Table 1:** List of primers used in qPCR for chondrogenic genes.

Genes	Primer sequences
*Col X*	F: GAACCCAGAATCCATCTGAG
	R: GGCATAGGGAATGAAGAACTG

*Sox9*	F: AGTAGGCAATAGTGTAGAGGAC
	R: CGGTGTTTAAGGCTCAAGG

*Col II*	F: CAAGTCCCTCAACAACCAG
	R: TATCCAGTAGTCACCGCTC

*Acan*	F: TGCCACTGTGAGAGTTCC
	R: ACATTCCACACCCAGAGTT

## Data Availability

All data generated or analyzed during this study are included in this article. Further inquiries can be directed to the corresponding author.
